# The fetomaternal interface in the placenta of three species of armadillos (Eutheria, Xenarthra, Dasypodidae)

**DOI:** 10.1186/1477-7827-10-38

**Published:** 2012-05-04

**Authors:** Lorenna C Rezende, Claudio G Barbeito, Phelipe O Favaron, Andrea Mess, Maria A Miglino

**Affiliations:** 1 Department of Surgery, Faculty of Veterinary Medicine, University of Sao Paulo, Av. Prof. Dr. Orlando Marques de Paiva, no. 87, Cidade Universitária, São Paulo, SP, CEP 05508-000, Brazil; 2 Faculty of Veterinary Science, La Plata University, 118 La Plata, Buenos Aires, Argentina

## Abstract

**Background:**

Placental characters vary among Xenarthra, one of four supraordinal clades of Eutheria. Armadillos are known for villous, haemochorial placentas similar to humans. Only the nine-banded armadillo has been well studied so far.

**Methods:**

Placentas of three species of armadillos were investigated by means of histology, immunohistochemistry including proliferation marker, and transmission and scanning electron microscopy.

**Results:**

The gross anatomy differed: *Euphractus sexcinctus* and *Chaetophractus villosus* had extended, zonary placentas, whereas *Chaetophractus vellerosus* had a disk. All taxa had complex villous areas within the maternal blood sinuses of the endometrium. Immunohistochemistry indicated the validity of former interpretations that the endothelium of the sinuses was largely intact. Tips of the villi and the columns entering the maternal tissue possessed trophoblast cell clusters with proliferation activity. Elsewhere, the feto-maternal barrier was syncytial haemochorial with fetal vessels near the surface.

**Conclusions:**

Differences among armadillos occurred in regard to the extension of the placenta, whereas the fine structure was similar. Parallels to the human suggest that armadillos are likely to be useful animal models for human placentation.

## Background

Molecular cladistics divides eutherian mammals into four supraordinal clades. Xenarthra likely has a basal position and may influence interpretations of character transformations [1-4]. Phylogenetics have stimulated evolutionary studies as on the placental system [5-9]. Distinct placental types have been recognized with regard to invasiveness, shape and internal organization [6,8]. However, only about 1% of eutherian species have been investigated, varying on ordinal and family levels [10]. Attention to rather exotic species such as armadillos were drawn from the middle of 20^th^ century on [11,12], indicating that placental characters vary within Xenarthra. Armadillos are known for haemochorial, villous placentas [11-14] similar to the human [15], indicating that they could be used as animal models [16]. Only the nine-banded armadillo *Dasypus novemcinctus* from North America is well studied in regard to placental development [11-14,17-20]. Data show a peculiar condition in that the developing villi entered pre-existing maternal blood sinuses and enlarged them while leaving the maternal vessels endothelium largely intact [11,13,14]. The sinuses were supplied by derivatives of the spiral arteries that were passing in endometrial septae and opened into the intervillous space [11,13,14]. However, data for other armadillo species are sparse [21-23]. We investigated armadillos from Brazil and Argentina, the large hairy armadillo *Chaetophractus villosus*desmarest, 1904, the small hairy armadillo *Chaetophractus vellerosus*gray, 1865, and the six-banded armadillo *Euphractus sexcinctus,*linnaeus, 1758*.* The species studied here exhibit litter sizes of 1 to 3 young and gestation periods of 60 to 70 days and inhabit pampas, cerrado and chaco vegetations [24-28].

## Methods

Term placentas of *Chaetophractus villosus* and *Chaetophractus vellerosus* were obtained from the Museum of the University of La Plata, Argentina. Two pregnant females of *Euphractus sexcinctus*, both in the second half of gestation, were obtained from wild specimens found dead on a Brazilian highway. Tissues were processed similar to former studies (see [29-31] for details). Material for histology, fixed in 10% formalin in 0.1 M phosphate buffer, was sectioned at 5 μm and stained with hematoxylin and eosin (HE), Masson´s Trichrome and periodic acid-Schiff reaction (PAS). Samples for SEM were fixed in 2.5% glutaraldehyde, post-fixed in 2% phosphate-buffered osmium tetroxide, critical point dried and gold sputtered. TEM samples were embedded in Spurr’s Resin; ultrathin sections were contrasted with 2% uranyl acetate and 0.5% lead citrate. We used TEM only for the feto-maternal barrier, because the quality of the material derived from museums and dead animals was not the best. Immunohistochemistry (see [29-31]) was performed in *Chaetophractus villosus* for vimentin (mouse monoclonal anti-human antibody: RTU-VimV9; ready to use concentration; novocastra, Wetzlar, Germany) and cytokeratin (rabbit polyclonal antibody: wide spectrum screening N1512; ready to use concentration; dako, Cytomation, Carpinteria, USA) and as a proliferation marker a mouse monoclonal antibody to human anti-PCNA (proliferation cell nuclear antigen, clone PC10; 1:300; sigma, St Louis, USA); then a biotinylated secondary antibody and streptavidin-HRP (Dako) were applied and detection was done by Envision and a DAB and substrate chromogen system (dako). Negative controls were performed using bovine serum albumin to substitute the primary antibody. The research was approved by the Ethical Committee at the Faculty of Veterinary Medicine and Animal Science of the University of Sao Paulo.

## Results

**Figure 1:**
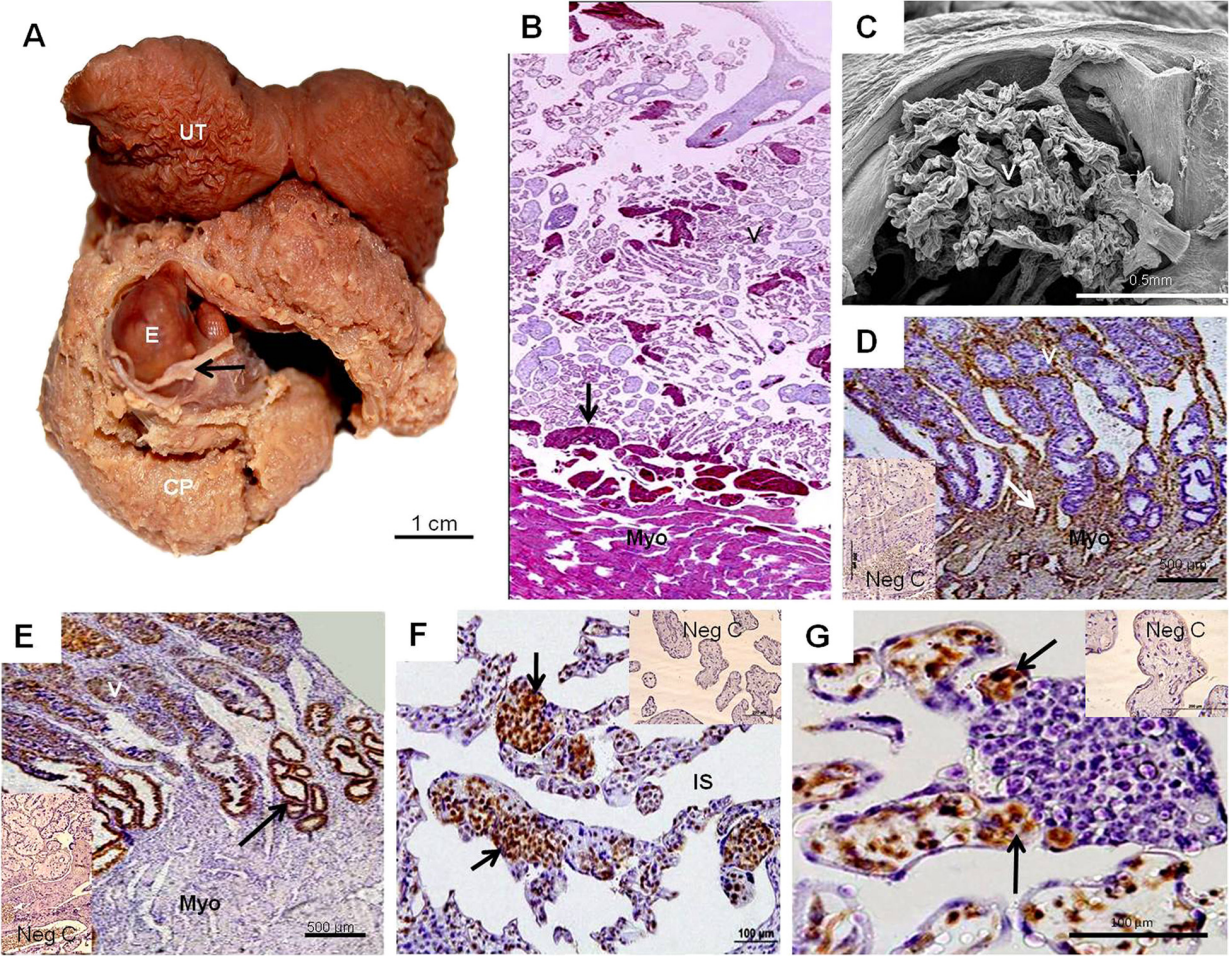
***Chaetophractus villosus***. (**A**) Macroscopic anatomy. Uterus (U) with extensive chorioallantoic placenta (CP) and a single embryo (E) covered by the amnion (arrow). (**B**) Hematoxylin and eosin. Montage of the villous area (V) above the myometrium (Myo) with trophoblast within venous spaces (arrow). Magnification 10*. (**C**) Scanning electron microscopy. Branching villi. (**D**) Vimentin. Tips of the villi insides maternal blood sinuses with remnants of vimentin-positive endothelium. Some glands were present (arrow). (**E**) Cytokeratin. Positive response in the trophoblast and in derivatives of the spiral arteries (arrows). (**F**) Proliferation cell nuclear antigen. Proliferation activity was high in the trophoblast cell clusters (arrow). (**G**) Hypertrophied mesenchymal cells that reacted positive to vimentin were present (arrow).

**Figure 2:**
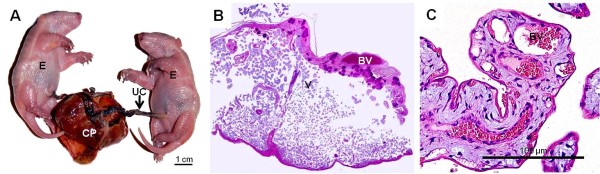
**Chaetophractus vellerosus**. (**A**) Macroscopic anatomy. Chorioallantoic placenta disk (CP) of two embryos (E) with separate umbilical cords (UC). (**B**) Hematoxylin and eosin. Montage of the villous area (V) with fetal blood vessels inside (BV). Magnification 4*. (**C**) Hematoxylin and eosin. Villi in the intervillous space with capillaries near the surface.

**Figure 3:**
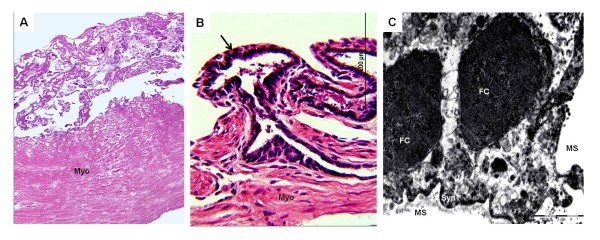
**Euphractus sexcinctus**. (**A**) Hematoxylin and eosin. Montage of the villous area (V) above the myometrium (Myo). Magnification 10*. (**B**) Hematoxylin and eosin. Fetomaternal interface with spiral artery derivative (arrow). (**C**) Transmission electron miscroscopy. A single-layered barrier with syncytial trophoblast (Syn T) was situated between the fetal capillaries (FC) and maternal blood spaces (MS).

The gross anatomy of the chorioallantoic placenta differed in the three armadillos: *Chaetophractus villosus* (Figure 1A) and *Euphractus sexcinctus* had extended, zonary placentas and *Chaetophractus vellerosus* had a disk (Figure 2A). *Chaetophractus villosus* had 3 fetuses within a single chorionic sac (Figure 1A); whereas *Chaetophractus vellerosus* (Figure 2A) and *E. sexcinctus* had two fetuses. The placentas of different embryos were not fully separated (Figure 2A). The placenta was established at the fundic end of the uterus. Independent from the macroscopic organization, all three taxa had complex villous areas within the maternal blood sinuses of the endometrium (Figures 1B,C,2B,3A) that reached the myometric region. Indicated by the application of vimentin and cytokeratin in *Chaetophractus villosus*, the blood sinuses retained parts of the endothelial border (Figures 1D,E). In all three species, this regions were supplied by derivatives of the spiral arteries that had been invaded by the trophoblast (Figures 1D,E,3B). These vessels run within connective tissue that was derived from the endometrium (Figure 2B). Tips of the villi and columns entering the maternal tissue possessed clusters of trophoblast cells (Figures 1B,D,E). Immunohistochemistry showed that they were active in proliferation (Figure 1F). The endometrial blood sinuses were confluent with the intervillous space. There, the feto-maternal barrier was syncytial haemochorial, one-layered and thin in places (Figures 1G,2C,3C). Vessels were situated close to the surface and hypertrophied mesenchymal cells occurred (Figures 1G,2C,3C).

## Discussion

Differences in armadillos have been revealed in regard to the form of the definitive placenta. Extended, zonary placentas occurred in *Chaetophractus villosus, Euphractus sexcinctus* as well as in *Cabassous chacoensisTolypeutes matacus* and *Dasypus hybridus*[22]. *Dasypus novemcinctus* possessed a restricted zonary placenta [11,13]. A disk occurred in *Chaetophractus vellerosus*. For *Chaetophractus villosusChaetophractus vellerosus* and the other species this represented the term state, whereas for *Euphractus sexcinctus* we did not have material from late gestation. All armadillo species studied so far had a complex villous area [11-14,17-23]. The application of immunohistochemistry in *Chaetophractus villosus* indicated the validity of former interpretations derived from *Dasypus novemcinctus*, that the developing villi entered maternal blood sinuses without fully destroying and replacing the vessels endothelium [11,13,14]. In addition, our results proved the expression of a proliferation marker in the trophoblast cell clusters especially occurring at the tips of the villi [11-13,19-23]. Thus, proliferation can be assumed as general activity of these cells, as was suggested by former work on morphology only [11-13]. In the human, the syncytiotrophoblast is formed from proliferative cytotrophoblast cells [14,32,33]. We suggest that these cytotrophoblast cells in armadillos may have a similar function. Elsewhere in the villi, the interhaemal barrier was haemomonochorial, syncytial and thin in places; these areas were associated with hypertrophied mesenchymal cells and capillaries that were near the surface [11-14,19-23]. These features exhibit parallels to humans [15]. The way in which the villous areas were established was different in primates and armadillos. In humans invasive trophoblast cells migrate deeply into the maternal tissues and remove the endothelium of the arteries [32-35]. However, armadillos are among the very few mammals with villous and invasive placentas. Thus, they may play a role as additional animal models for human placentation [16].

## Conclusions

Principal differences between three armadillo species have been revealed in regard to the extension of the placenta, whereas the fine structure was mostly similar. Major parallels occurred compared to the human, suggesting that armadillos may play a role as additional animal models for particular questions.

## Competing interests

The authors declare that they have no competing interests.

## Authors' contributions

MAM devised the study and participated in its design. LCR established the procedure of the material and performed the analysis, helped by CGB, SASK and POF. AM wrote the manuscript. All authors read and approved the final manuscript.
